# Use of a Therapeutic Trial of Graduated Neoadjuvant Radiation Therapy for Locally Advanced Esophageal Cancer in a Patient With Fanconi Anemia

**DOI:** 10.1016/j.adro.2021.100810

**Published:** 2021-09-29

**Authors:** Ulysses G. Gardner, Stephanie G. Wood, Emerson Y. Chen, Joel S. Greenberger, Aaron J. Grossberg

**Affiliations:** a Department of Radiation Oncology and Molecular Radiation Sciences, The Johns Hopkins University School of Medicine, Baltimore, Maryland; b Department of Surgery, Oregon Health and Science University, Portland, Oregon; c Division of Hematology and Medical Oncology, Department of Medicine, Knight Cancer Institute, Oregon Health and Science University, Portland, Oregon; d Department of Radiation Oncology, The University of Pittsburgh School of Medicine, Pittsburgh, Pennsylvania; e Department of Radiation Medicine, Knight Cancer Institute, Oregon Health and Science University, Portland, Oregon

## Introduction

Fanconi anemia (FA) is a rare and inherited disorder leading to an inability to repair DNA by homologous recombination.^1^^,^^2^ The product of the FA pathway is DNA stability through interstrand crosslink repair.^3^ A loss of function in the FA pathway causes cell hypersensitivity to genotoxic stress, results in a loss of hematopoietic stem cells, and increases the predilection to certain malignancies.^3^, ^4^, ^5^

The presentation of FA is highly genotypically and phenotypically heterogenous. Manifestations include aplastic anemia, birth defects, physical abnormalities, and endocrine dysfunction.^6^, ^7^, ^8^ FA is strongly associated with the development of malignancies, specifically of the esophagus, vulvar, and head and neck region.^9^^,^^10^ Squamous cell carcinoma (SCC) of the head and neck region is the most common solid tumor in patients with FA with up to a 700-fold increase in relative risk.^11^^,^^12^

With improvements in hematopoietic stem cell transplantation using fludarabine-based, reduced-intensity conditioning, low-dose or partial-body irradiation, and the improved management of complications caused by pancytopenia, patients are living into their fourth and fifth decade of life with FA.^13^^,^^14^ Bone marrow transplantation, the only known cure for bone marrow failure, is required in 90% of cases of FA; however, the risk of developing head and neck, esophageal, gastrointestinal, vulvar, and anal cancers is significantly higher at approximately 50-fold.^15^

The standard of care for clinically resectable, locally advanced, squamous cell esophageal carcinoma is concurrent chemotherapy and radiation therapy (CRT) followed by resection. In the CROSS (ChemoRadiotherapy for Oesophageal cancer followed by Surgery Study) trial, a prospective randomized trial comparing neoadjuvant chemoradiation (carboplatin and paclitaxel; 41.4 Gy in 23 fractions) followed by surgery versus surgery alone, the addition of neoadjuvant CRT improved median overall survival (OS; 49.4 months vs 24.0 months in the surgery alone arm) and complete resection (R0) rate (92% vs 69% in the surgery alone arm). A pathologic complete response (pCR) was achieved in 29% of patients after resection after CRT.^16^ These effects were more pronounced for patients with SCC, in whom median OS was 81.6 (95% confidence interval [CI], 47.2-116.0) months in the neoadjuvant plus surgery group versus 21.1 (95% CI, 15.4-26.7) months in the surgery alone group, and pCR was observed in 49%.^17^ In patients with FA, some of whom display a defective repair of DNA crosslinks and double-strand breaks, the application of CRT creates a therapeutic challenge. Case reports reveal that radiation stimulates more severe toxicities in patients with FA than the general population, including mucositis and life-threatening pancytopenia.^18^, ^19^, ^20^

Although the associated risk of using CRT is excessive in patients with FA, the magnitude of survival benefit offered by the CROSS regimen makes a compelling case for neoadjuvant therapy. However, we have no means of risk stratification to determine for which patients the benefit of neoadjuvant therapy outweighs the risk. Recently, a definitive RT approach involving graduated escalation of fractional dose and volume in a patient with FA and head and neck cancer was successfully pioneered.^21^ A trial dose was first administered to each successively larger volume with close monitoring for toxicity before escalating to conventional RT fractions, a concept introduced for myeloid metaplasia.^22^ Although no analogous reports were found for esophageal cancer, we reasoned that a similar approach could be used in a patient with FA who developed esophageal SCC.

## Case

Our patient was a 26-year-old, relatively healthy man diagnosed with FA at age 8. He underwent total-body irradiation with conditioning before an allogenic stem cell transplant at age 11 complicated by mild graft versus host disease for which he was treated with immunosuppression for <6 months. His complete blood cell count and graft function has been normal since that time. He presented with complaints of a lump in his throat and intermittent dysphagia.

**Figure 1:**
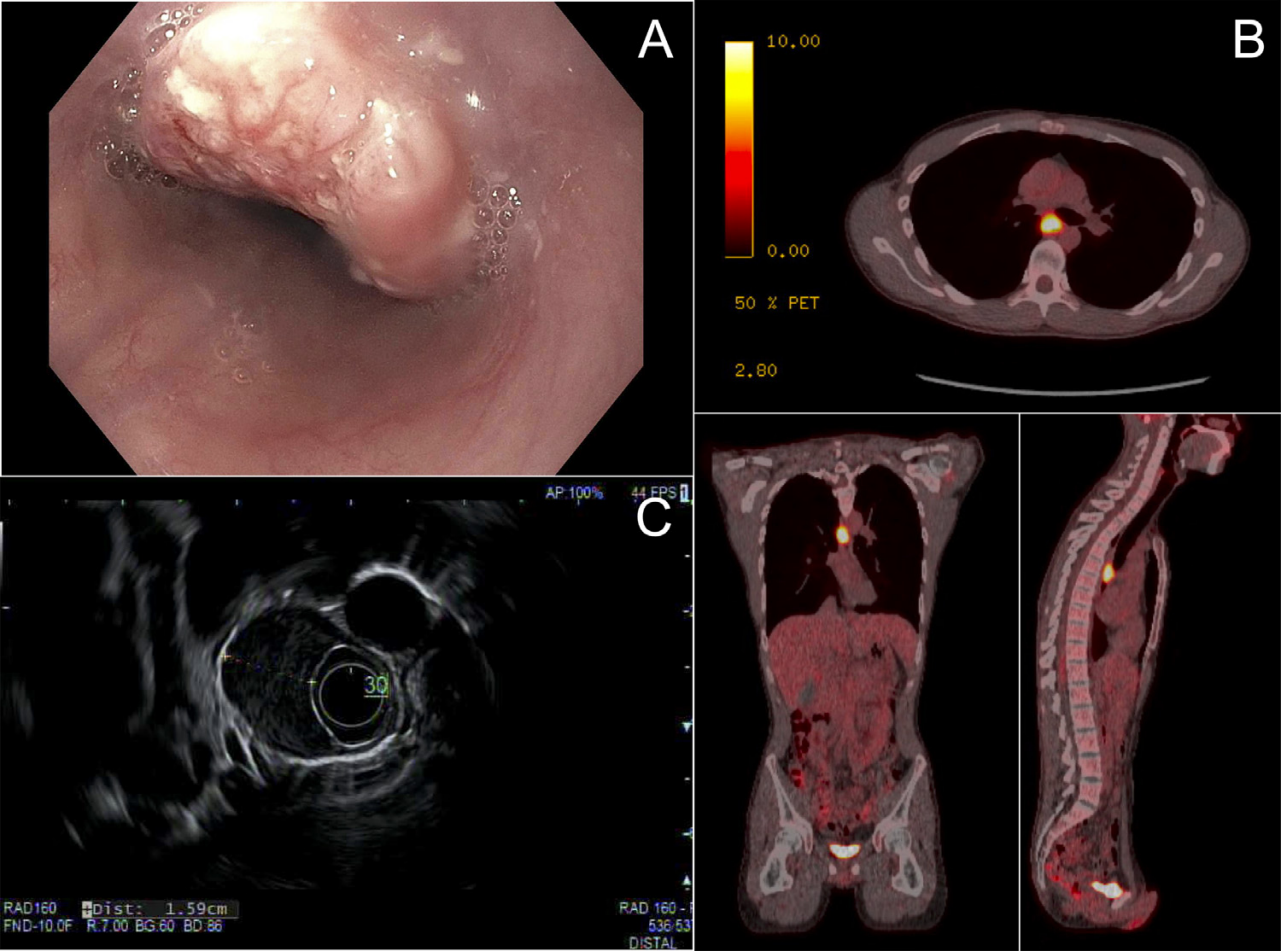
Staging at time of diagnosis. (A) Esophagogastroduodenoscopy revealing a friable, ulcerating nonobstructive mass in the proximal esophagus extending 25 cm to 28 cm from the incisors, obstructing about 50% of the esophageal lumen. (B) Positron emission tomography/computed tomography showing fluorodeoxyglucose-avid mass in the midesophagus (SUV max = 15.6). No fluorodeoxyglucose-avid lymph nodes or distant metastases were visualized. (C) Endoscopic ultrasound of midesophageal mass measuring 16 mm in thickness invading into the muscularis propria.

On esophagogastroduodenoscopy (EGD), he was noted to have a large, friable, ulcerating nonobstructive mass extending 25 to 28 cm from the incisors (Fig. 1A). The entire examined stomach and first and second portions of the duodenum were normal. Biopsy revealed invasive SCC, moderately differentiated. Computerized tomography (CT) revealed a 16 mm esophageal mass at the level of the left mainstem bronchus without metastatic disease. Positron emission tomography/CT demonstrated a fluorodeoxyglucose-avid midesophageal mass. No nodal or distant metastatic disease was noted (Fig. 1B). Endoscopic ultrasound revealed a hypoechoic mass in the middle third of the esophagus, involving 50% of the lumen, and measured 16 mm in thickness with evidence of invasion into the muscularis propria (Fig. 1C). One 3 × 2 mm, oval lymph node was visualized in the middle paraoesophageal mediastinum (level 8M) indicating stage uT2N1.

**Figure 2:**
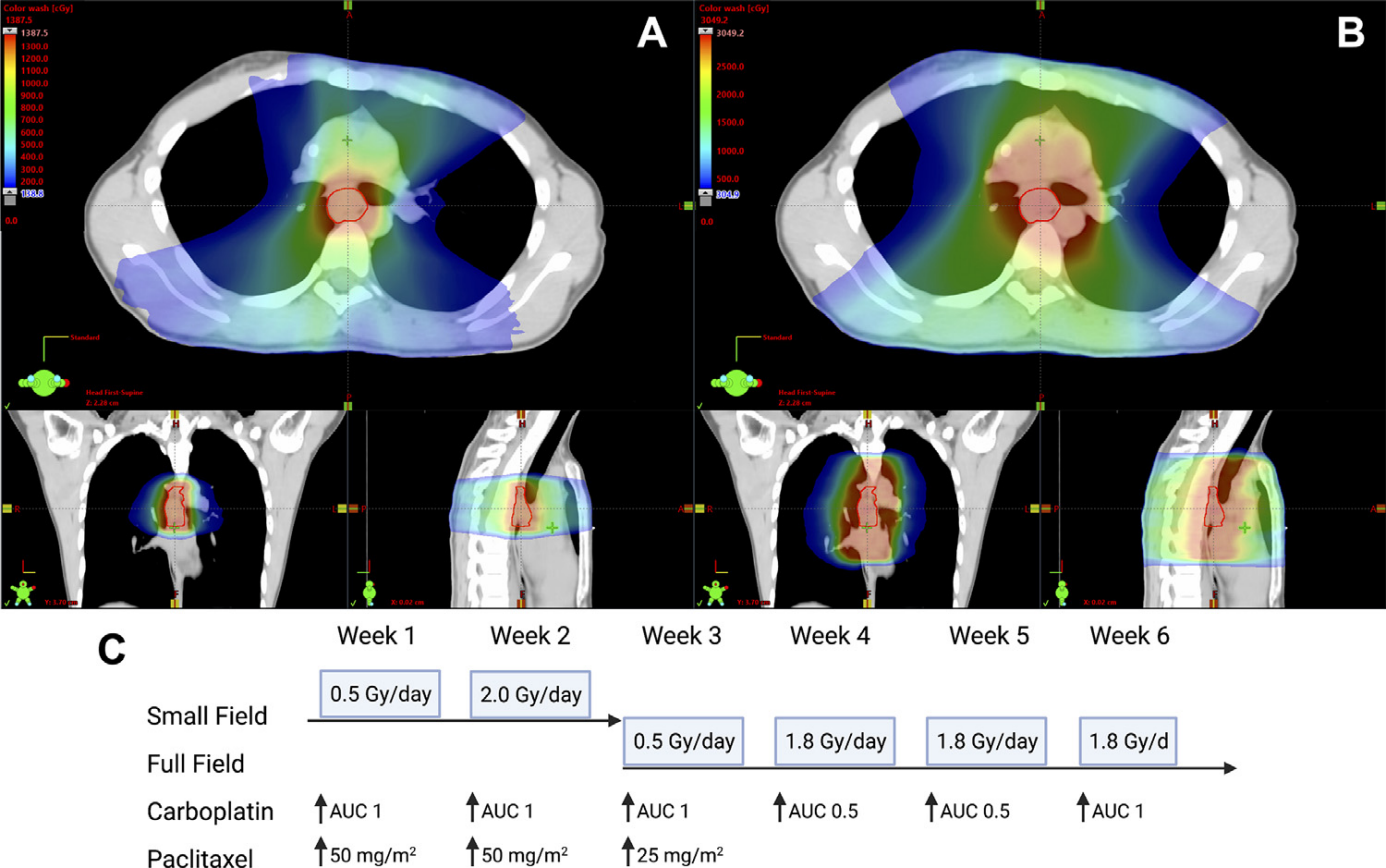
Neoadjuvant treatment delivery. (A) Images of the dose distribution of the small field IMRT plan. Radiation therapy delivered to the small field for 5 days at 0.5 Gy/d and then at 2 Gy/d for another 5 days. (B) Dose distribution to the full field, delivered over 4 weeks. Full field was treated for 5 days at 0.5 Gy/d, then at 1.8 Gy/d for a subsequent 14 fractions over 3 weeks. (C) Treatment schedule, including chemotherapy timing and dose. Carboplatin was delivered weekly at area under the curve 1 initially, with dose reduction to area under the curve 0.5 for cycles 4 and 5. Paclitaxel was administered at 50 mg/m^2^ for first 2 cycles, then dose reduced to 25 mg/m^2^ for third cycle, before being omitted from cycles 4 to 6. (A and B) Images shown in the axial (top), coronal (bottom left), and sagittal (bottom right) planes.

Multidisciplinary discussion weighed upfront surgery against neoadjuvant CRT, out of concern for a greater chance for CRT-associated high-grade toxicity. Given the dramatic improvement in survival with neoadjuvant therapy, a modified CROSS technique was agreed on by the oncologists, hematologists, and surgeons in collaboration with physicians from the Fanconi Anemia Research Fund. Platinum doublets, the backbone of neoadjuvant chemotherapy in esophageal cancer, derive their antitumor activity by generating platinum-DNA adducts leading to interstrand crosslinking. Because FA is associated with impaired repair of such crosslinks, his plan included weekly dose-reduced carboplatin area under the curve (AUC 1) (50% dose reduction) plus paclitaxel 50 mg/m^2^ (standard dose) (Fig. 2).

Radiation was planned as a therapeutic trial of graduated doses and volumes, using a 4-stage approach. The patient was monitored for toxicities daily and weekly for cytopenia. Treatment was delivered via intensity modulated radiation therapy with daily cone beam CT. A planning target volume (PTV) measuring 36.5 cm^3^ was first treated to a dose of 0.5 Gy per fraction for the first 5 fractions (Fig. 2A), with the intention of aborting RT if he developed any symptoms during the low dose trial. In the second stage, we escalated the dose to the same volume to 2 Gy per fraction. During the third stage, a standard esophageal RT field, contoured in accord with consensus guidelines,^23^ was treated to 0.5 Gy in a trial of the larger volume (Fig. 2B). In the fourth stage, the full PTV was treated to 1.8 Gy per fraction for 14 fractions. During stages 2 to 4, esophageal toxicity was expected, and Common Terminology Criteria for Adverse Events version 5.0, grade 3 esophagitis (severe altered eating/swallowing; tube feeding, total parenteral nutrition, or hospitalization indicated) was selected a priori as the RT cessation trigger, although clinician judgment should be to terminate sooner if symptoms rapidly escalate. The total dose delivered in 29 fractions was 40.2 Gy (54 biologically effective dose [BED], α/β = 4.9) to the GTV with margin and 27.7 Gy in 19 fractions (37 BED, α/β = 4.9) to the full PTV (Fig. 1).

The first dose of chemotherapy was administered 1 day before the beginning of RT. He reported mild oral mucositis without dysphagia or odynophagia during the first week. At the end of his second week, he reported grade 2 oral mucositis and nausea; nausea managed with metoclopramide, prochlorperazine, and Zofran. For his third cycle, the paclitaxel was reduced to 25 mg/m^2^ (Fig. 2C). During week 3, he developed grade 3 nausea and vomiting with worsening of his baseline dysphagia requiring intravenous fluid resuscitation. Cycle 4 carboplatin was reduced to AUC 0.5 and paclitaxel discontinued secondary to myalgias, infusion reaction, and nausea and vomiting (Fig. 2C). His toxicities improved, reporting only increasing globus sensation and mild esophageal burning. During week 5, he developed grade 2 esophagitis. For cycle 6, carboplatin was increased back to AUC 1 and tolerated well, whereas the paclitaxel was withheld (Fig. 2C). Esophagitis was largely unchanged from the prior week and managed with hydrocodone and viscous lidocaine as needed. Hospital admission was not required at any time.

His complete blood counts remained acceptable throughout the treatment course. Of note, his baseline counts indicated his blood counts were derived from his allogeneic donor. Red blood cells averaged 4.71 M/cu mm (range, 4.28-5.21). Platelet count remained stable with a mean of 206 K/cu mm (range, 186-266). White blood cells generally decreased throughout treatment, averaging 5.38 K/cu mm (range, 3.43-8.36), with a nadir during week 6. If he presented before transplantation or had existing cytopenias, his complete blood cell count would have been checked at minimum 3 times a week.

**Figure 3:**
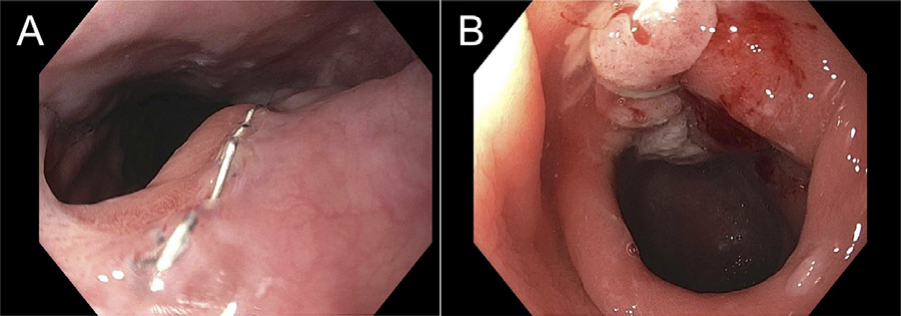
Postoperative esophagogastroduodenoscopy. Esophagogastroduodenoscopy obtained 18 weeks after surgery. (A) Normal, widely patent esophagogastric anastomosis with 4 small staples visible. Staples were removed and biopsy taken that showed chronic inflammation and no dysplasia or malignancy. (B) Localized mild mucosal changes characterized by granularity and nodularity at the widely patent pyloromyotomy site.

A positron emission tomography/CT scan obtained at 7 weeks after CRT demonstrated no metabolic active in the midesophagus. At 11 weeks, a minimally invasive 3-field esophagectomy with pyloromyotomy and intraoperative EGD revealed no evidence of residual tumor. Pathology indicated no residual esophageal carcinoma with negative margins and lymph nodes. An EGD performed 18 weeks after surgery revealed normal, widely patent esophagogastric anastomosis, normal stomach, and granular and nodular mucosa at the pyloromyotomy site, which was not biopsied due to elevated risk of full-thickness injury (Fig. 3). Esophagogastric anastomosis was biopsied, revealing squamous mucosa with only chronic inflammation. His final pathology was reviewed in multidisciplinary tumor board with recommendations for ongoing surveillance without additional adjuvant treatment. Nivolumab was not considered as he had a pCR and studies report severe toxicities in patients treated with programmed cell death-1 inhibitors after allogenic stem cell transplants.^24^, ^25^, ^26^ Institutional approval and written informed consent from the patient was obtained for publication of the case.

## Discussion

The incidence of developing an esophageal malignancy in FA patients is reportedly up to 6000 times higher than the general population.^9^^,^^10^^.^^27^ Case reports indicate esophageal cancers arise in FA patients in a much younger cohort, typically 20s to 30s years of age,^28^^,^^29^ highlighting the importance of a high clinical suspicion and early screening and detection.

Due to the instability of the FA pathway, chemotherapy and radiation therapy treatments are traditionally avoided in patients with FA to prevent adverse reactions and possible fatal complications. However, the phenotypes of FA patients are highly heterogenous and some FA variants may allow tolerance to chemotherapeutics and radiation. One case report describing an attempt to predict radiosensitivity in a patient with FA found a marked discrepancy between cellular and clinical radiosensitivity, indicating that in vitro radiosensitivity assays are unlikely to exhibit clinical utility.^30^

The use of a therapeutic trial is an approach that can be used to identify patients with FA who may tolerate therapeutic doses of RT. This technique introduces a low test dose of radiation in a small volume. As the pilot dose is tolerated, gradual escalation in treatment volume and dose is allowed with the intent of treating with a standard dose. In the case presented, no RT-associated acute grade 3 + toxicities were observed, and no late effects of radiation are appreciated now, 7 months after RT. Despite receiving a slightly lower BED than used in the CROSS (57 BED, α/β = 4.9) and dose-reduced chemotherapy, he achieved pCR. Due to the high risk of recurrence or new tumor development, we will continue to observe patients with FA with imaging.

It is important to note that the patient had undergone a successful bone marrow transplant, which likely contributed to his chemotherapy tolerance. Patients who have not had transplant are far more susceptible to cytopenias, requiring much closer surveillance. Recommendations advise against cytotoxic chemotherapy in patients treated for squamous cell carcinoma of the head and neck who have not undergone transplant, with a high frequency of cytopenias and early therapy cessation.^31^ We similarly caution against cytotoxic chemotherapies in untransplanted patients with esophageal cancers. Alternative approaches for these patients would include neoadjuvant radiation therapy alone, radiation therapy with concurrent antiepithelial growth factor receptor therapy^32^^,^^33^ or antiprogrammed cell death ligand-1 therapy for patients with a Combined Positive Score ≥10.^34^ These treatments have each been investigated in esophageal cancers with equivocal results; however, subgroup and pathologic analyses suggest benefit for patients with squamous histology.

## Conclusion

The RT plan reported herein allowed the full extent of gross disease to receive comparable total dose in this patient with minimal regional involvement. Patients whose nodal disease is not directly apposed to the primary tumor present a greater clinical challenge that might require treating multiple volumes to ensure adequate dose, if the distance between gross nodal disease and primary is large enough. A graduated therapeutic trial approach allows patients with FA the benefit of improved oncologic outcomes associated with neoadjuvant treatment for esophageal SCC, while providing the opportunity to avoid potentially life-threatening complications.
